# Upregulation of Peripheral Blood NLRP3 and IL‐18 in Patients With Acute Kidney Injury in Sepsis and Its Clinical Significance

**DOI:** 10.1002/iid3.70113

**Published:** 2024-12-18

**Authors:** Jing Zhou, Yibin Ye, Zhipeng Chen, Yong Liu, Baozheng Wu, Haiping Huang

**Affiliations:** ^1^ Department of Critical Care Medicine Zhangzhou Affiliated Hospital of Fujian Medical University Zhangzhou Fujian China; ^2^ Department of cerebrovascular Intervention Intensive Care Unit Zhangzhou Affiliated Hospital of Fujian Medical University Zhangzhou Fujian China

**Keywords:** GEO database, IL‐18, NLRP3, sepsis‐associated AKI

## Abstract

**Background:**

Sepsis‐associated acute kidney injury (SA‐AKI) is a common complication that can lead to renal failure in patients, significantly affecting the prognosis and survival of patients.

**Objective:**

In this study, we aimed to evaluate the predictive value of NOD‐like receptor protein 3 (NLRP3) and interleukin 18 (IL‐18) in peripheral blood mononuclear cells (PBMCs) of SA patients for the occurrence of SA‐AKI.

**Material and Methods:**

We screened AKI‐related data sets using the Gene Expression Omnibus (GEO) database and identified differentially expressed genes (DEGs) associated with AKI. KEGG and GO analysis were used to identify enriched molecular functions and pathways. The study included 62 SA patients admitted to the Department of Intensive Care Medicine of our hospital from February 2021 to June 2022, including 34 non‐AKI cases and 28 AKI cases, and 25 healthy volunteers were used as the control group. Real‐time quantitative polymerase chain reaction (RT‐qPCR) was used to detect the levels of NLRP3 and IL‐18 in PBMCs of the subjects.

**Results:**

Bioinformatics analysis and experimental validation showed that the expression levels of NLRP3 and IL‐18 were significantly upregulated in SA‐AKI patients. In addition, the expressions of NLRP3 and IL‐18 were positively correlated with APACHE II scores. ROC curve analysis revealed that NLRP3 and IL‐18 have the potential to diagnose SA‐AKI.

**Conclusion:**

This study provides preliminary evidence for NLRP3 and IL‐18 as potential diagnostic biomarkers for SA‐AKI.

## 1 Introduction

Sepsis is a systemic inflammatory response syndrome that can result from infection at any site [1]. Acute kidney injury (AKI) is one of the most common complications of sepsis, and the overall incidence of AKI in patients with sepsis in the intensive care unit (ICU) is about 15%–20% [2]. The occurrence of sepsis‐associated AKI (SA‐AKI) may lead to renal failure, seriously affecting the prognosis and survival of patients [3, 4].

The pathogenesis of SA‐AKI remains poorly understood, and its pathophysiology is characterized by a complex and multifactorial process [5]. Accumulating evidence indicates that inflammation plays a crucial role in renal tissue injury and systemic inflammation [6]. The inflammatory response triggered during SA‐AKI may promote the activation of the inflammasome. The inflammasome is a complex formed by multiple proteins, of which the NOD‐like receptor protein 3 (NLRP3) inflammasome is the most extensively studied [7]. The activated inflammasome triggers a cascade of responses, including the production of pro‐inflammatory cytokines such as interleukin‐1β (IL‐1β) and interleukin‐18 (IL‐18). These responses significantly exacerbate kidney cell damage and trigger an inflammatory cascade. However, studies on the association of NLRP3 and IL‐18 with the occurrence of SA‐AKI are still limited.

In this study, bioinformatics tools were applied to screen key genes associated with SA‐AKI. The AKI sequencing data set GSE61739 downloaded from the GEO was performed for secondary analysis, and it was found that the levels of NLRP3 and IL‐18 were upregulated in the AKI group compared with the non‐AKI group. We hypothesized that NLRP3 and IL‐18 may be the key genes in the pathogenesis of SA‐AKI. We then investigated the expression level of NLRP3 and IL‐18 in peripheral blood mononuclear cells (PBMCs) of SA‐AKI patients and analyzed the correlation of NLRP3 and IL‐18 with the occurrence of SA‐AK. The purpose of this study was to provide preliminary evidence on whether NLRP3 and IL‐18 have potential as diagnostic biomarkers for SA‐AKI.

## 2 Materials and Methods

### 2.1 Data Acquisition and Differential Gene Screening

Data sets were downloaded from the GEO database. In the GSE61739 data set, the gene expression profiles of kidney tissues from 24 AKI patients and 24 non‐AKI patients were compared. The differential gene expression was analyzed using the edgeR package (*p* < 0.05, |log FC | ≥ 1). The ggplot2 package was used to draw the volcano map, and the clusterProfiler package was used for GO function enrichment analysis and KEGG pathway analysis.

### 2.2 Clinical Samples

From February 2021 to June 2022, 62 patients with sepsis admitted to the ICU of Zhangzhou Hospital Affiliated to Fujian Medical University were enrolled. According to the occurrence of AKI, patients with sepsis were divided into two groups, AKI group and non‐AKI group. In addition, 25 healthy volunteers without sepsis were selected as the control group. The sepsis cases in this study were diagnosed according to the Sepsis 3.0 guidelines [8]. Diagnosis of AKI based on KDIGO criteria [9]. The research protocol was approved by the Ethics Committee of Zhangzhou Hospital Affiliated to Fujian Medical University (approval number: 2021KYB017). All participants signed the informed consent in advance.

### 2.3 Quantitative Real‐Time PCR

A 5 mL fasting blood sample was collected from each patient transferred to the ICU the next morning, and volunteers in the control group were drawn during physical examination. PBMCs were isolated by whole blood. Total RNA was extracted from PBMCs using Trizol method. The following steps were performed strictly according to the PCR kit manufacturer's instructions (Takara, Otsu, Japan). GAPDH served as an internal benchmark. The relative mRNA expression of NLRP3 and IL‐18 was calculated using the 2^‐Δ*Ct*^ method. The primer sequences relevant to the study are included in Table 1.

**Table 1:** Sequences of RT‐PCR primers.

Gene name	Primer	Sequence 5′ > 3′
GAPDH	Forward	GTCTCCTCTGACTTCAACAGCG
	Reverse	ACCACCCTGTTGCTGTAGCCAA
NLRP3	Forward	GGACTGAAGCACCTGTTGTGCA
	Reverse	TCCTGAGTCTCCCAAGGCATTC
IL‐18	Forward	GATAGCCAGCCTAGAGGTATGG
	Reverse	CCTTGATGTTATCAGGAGGATTCA

### 2.4 Statistical Analysis

SPSS v. 25.0 (SPSS Inc., Chicago, USA) and GraphPad Prism v. 7.0.1 (GraphPad Software, San Diego, USA) were used for statistical analysis. Quantitative results were expressed as mean ± standard deviation. For intergroup comparisons of quantitative data, we employed the Mann–Whitney *U* test. Categorical data were presented as frequencies and proportions (*n*, %), with group comparisons performed using the Pearson chi‐square (*χ*²) test. ROC curve was used to evaluate the diagnostic value of NLRP3 and IL‐18 among different groups. Pearson analysis was used for correlation analysis. *p* < 0.05 was considered statistically significant.

## 3 Results

### 3.1 Identification of DEGs Between AKI Patients and Non‐AKI Patients

**Figure 1:**
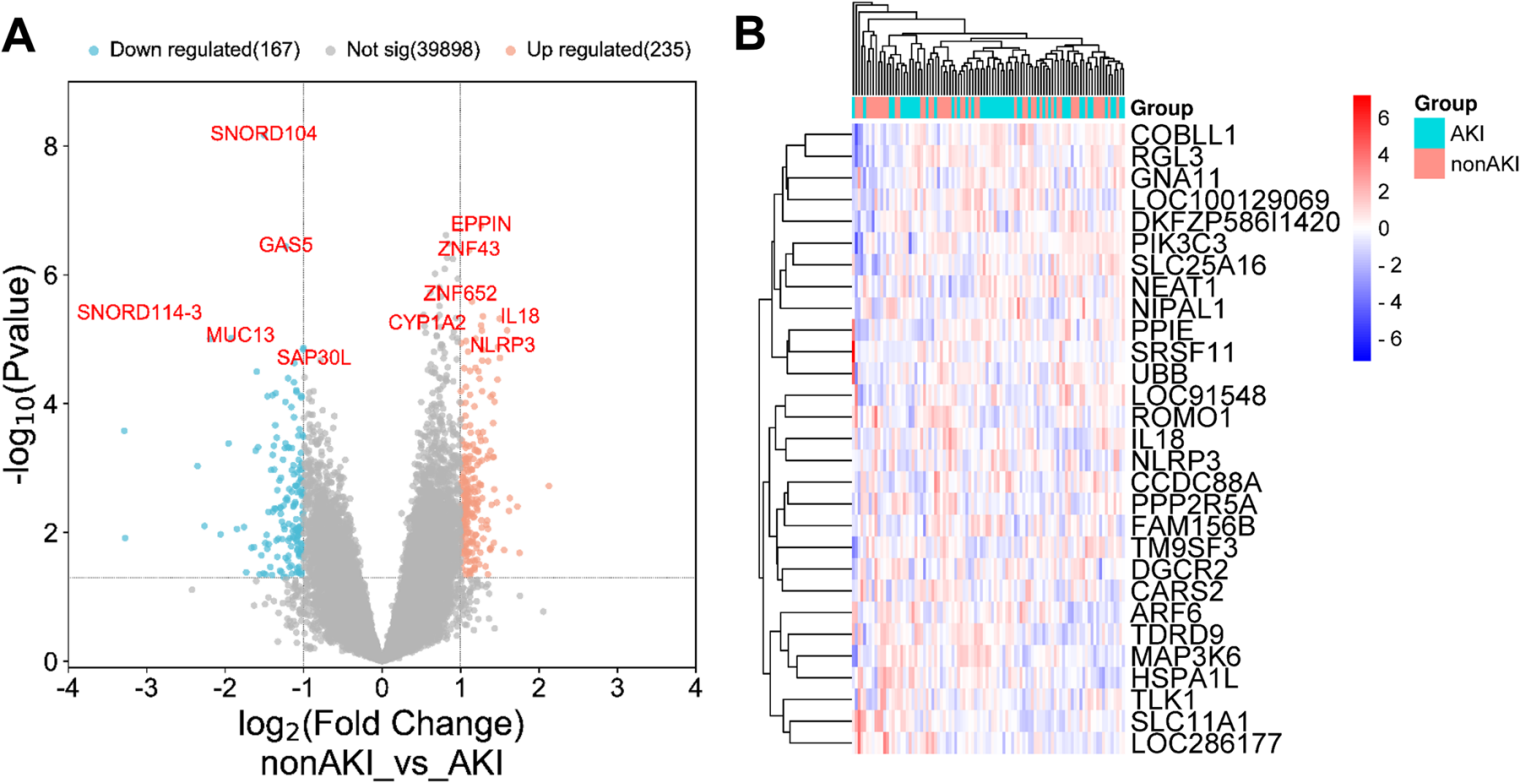
DEGs screened from GEO expression profiles associated with AKI. (A) Volcano map of DEGs. (B) Heat map of DEGs.

**Figure 2:**
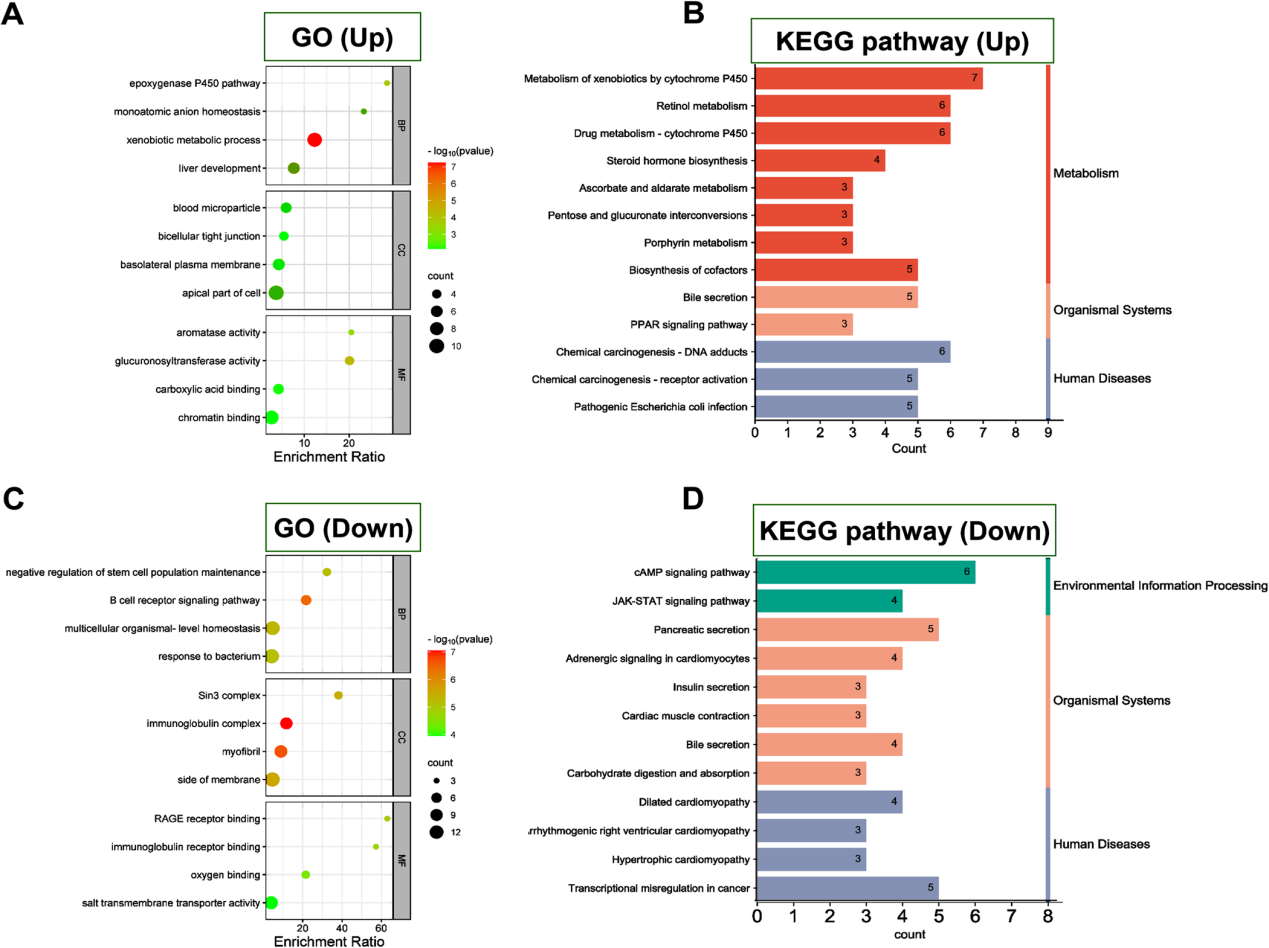
KEGG/GO analysis of 235 upregulated and 167 downregulated DEGs. GO (A) and KEGG enrichment analyses (B) of upregulated genes. GO (C) and KEGG enrichment analyses (D) of downregulated genes.

The GSE61739 data set revealed 402 DEGs between AKI patients and non‐AKI patients. Among them, 235 genes were upregulated, while 167 genes were downregulated, as shown in Figure 1. GO enrichment analysis showed that upregulated genes were mainly related to exogenous metabolism, and downregulated genes were mainly related to bacterial responses (Figure 2A,C). KEGG pathway analysis showed that upregulated genes were mainly concentrated in biological metabolic pathways, and downregulated genes were mainly concentrated in the organismal system (Figure 2B,D). These findings contribute to our understanding of the molecular mechanisms underlying AKI.

### 3.2 Characteristics of the Study Population

Among the 62 ICU patients diagnosed with sepsis, none had developed AKI at the time of admission. However, 28 of these patients subsequently developed SA‐AKI. The average time from ICU admission to the onset of AKI was 2.75 days. When comparing the non‐AKI and AKI groups, there were no statistically significant differences in terms of age (*p* = 0.748), gender (*p* = 0.180), body mass index (BMI, *p* = 0.346), baseline blood urea nitrogen (BUN, *p* = 0.163), or the duration of ICU stay (*p* = 0.605). However, the AKI group exhibited significantly higher levels of both baseline and peak serum creatinine (Scr, *p* = 0.001; *p* < 0.001), peak BUN (*p* < 0.001), and APACHE II scores (*p* < 0.001), along with a significantly reduced estimated glomerular filtration rate (eGFR, *p* < 0.001). Detailed data are presented in Table 2.

**Table 2:** Demographic and clinical characteristics of the study subjects.

Parameter	Control group (*n* = 25)	non‐AKI group (*n* = 34)	AKI group (*n* = 28)	*p*‐value
Age (years)	74.76 ± 14.50	75.09 ± 14.60	73.93 ± 13.43	0.748
Gender, male, (*n*, %)	14 (56.00)	26 (76.47)	17 (60.71)	0.180
BMI (kg/m^2^)	21.31 ± 1.38	20.04 ± 1.88	19.59 ± 1.84	0.346
Base line Scr (μmol/L)	70.19 ± 14.73	69.63 ± 24.22	150.09 ± 118.65	0.001
Peak Scr (μmol/L)	—	74.60 ± 22.25	276.19 ± 154.35	< 0.001
Base line BUN (mmol/L)	5.71 ± 1.87	10.52 ± 10.57	13.03 ± 9.66	0.163
Peak BUN (mmol/L)	—	9.34 ± 5.39	25.03 ± 14.35	< 0.001
eGFR (mL/min/1.73 m²)	63.33 ± 19.06	62.61 ± 23.52	18.56 ± 8.13	< 0.001
APACHE Ⅱ score	—	16.79 ± 1.36	26.21 ± 8.51	< 0.001
Days from ICU Admission to AKI (days)	—	—	2.75 ± 1.32	—
Days in the ICU (days)	—	8.88 ± 6.60	10.14 ± 7.92	0.605

*Note:* The *p*‐value represents a comparison between the non‐AKI group and the AKI group.

Abbreviations: “—,” not applicable; APACHE Ⅱ score, acute physiology score and acute physiology and chronic health evaluation; BMI, body mass index; BUN, blood urea nitrogen; eGFR, estimated glomerular filtration rate; ICU, intensive care unit; Scr, serum creatinine.

### 3.3 NLRP3 and IL‐18 Were Upregulated Consistent With Bioinformatics Analysis

**Figure 3:**
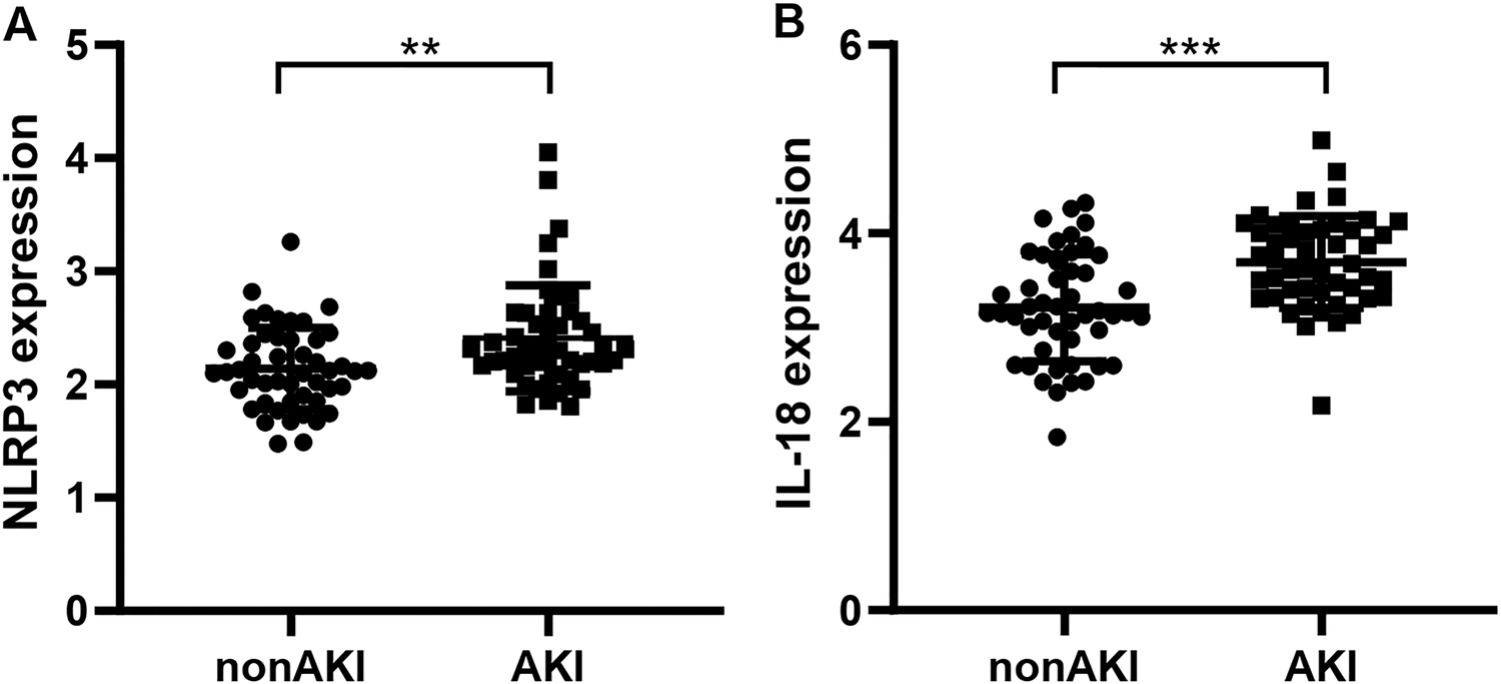
Expression analysis of NLRP3 and IL‐18 in GEO expression profiles. (A) NLRP3 expression. (B) IL‐18 expression. **p < .01, ***p < .001 versus non‐AKI.

**Figure 4:**
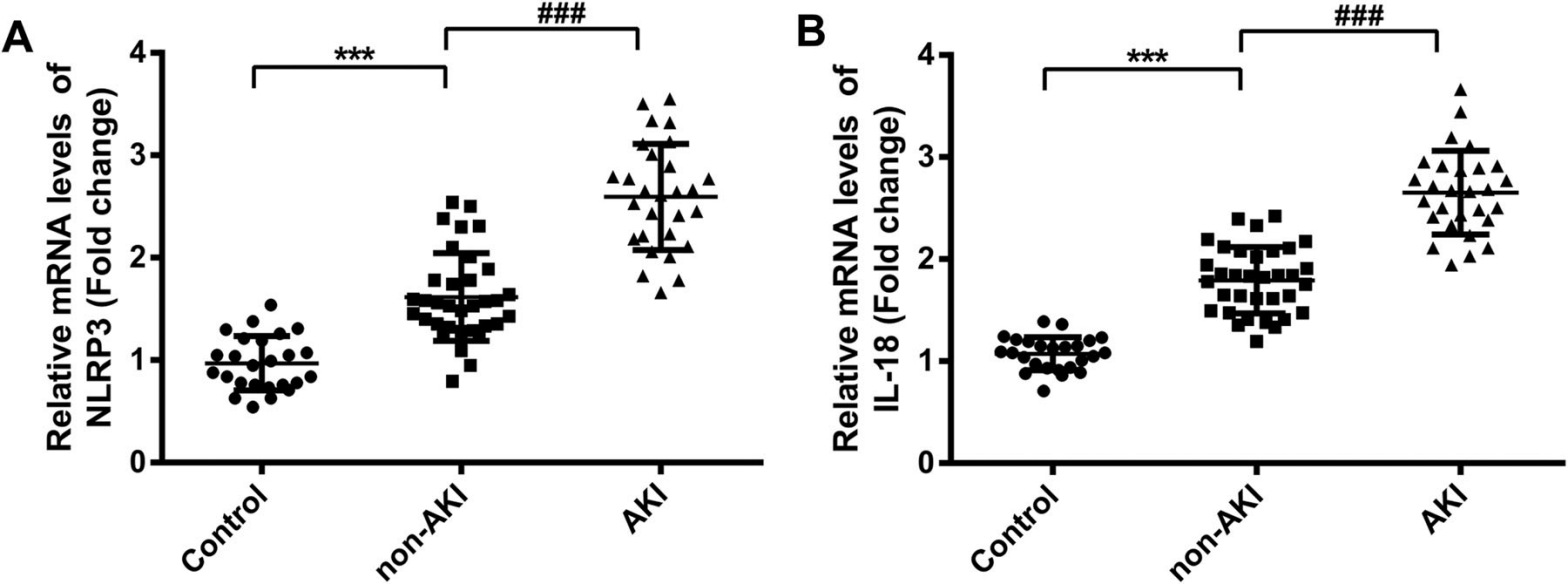
NLRP3 and IL‐18 expressions were elevated in PBMCs from SA‐AKI patients. (A) The mRNA level of NLRP3 was assessed by RT‐qPCR. (B) The mRNA level of IL‐18. ***p < .001 versus control; ^###^ *p* < .001 versus non‐AKI.

We selected NLRP3 and IL‐18 genes that were significantly upregulated in AKI patients for further study. Bioinformatics results showed that the expressions of NLRP3 and IL‐18 were significantly increased in AKI patients (*p* < 0.01; *p* < 0.001) (Figure 3). The mRNA levels of NLRP3 and IL‐18 in PBMCs of AKI patients were detected by RT‐qPCR, and the results showed that compared with non‐AKI group, NLRP3 and IL‐18 in PBMCs of AKI group were significantly upregulated (*p* < 0.001; *p* < 0.001) (Figure 4).

### 3.4 The Expressions of NLRP3 and IL‐18 Were Positively Correlated With APACHE II Score

**Figure 5:**
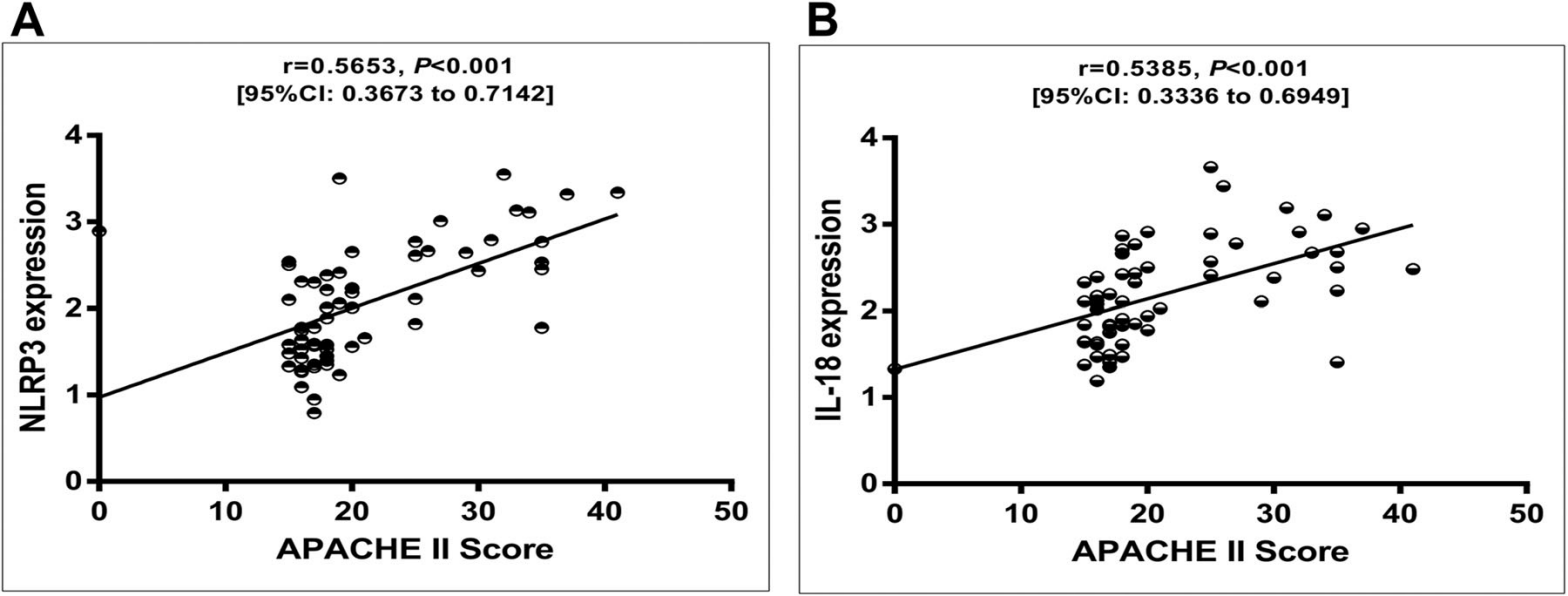
Correlation between NLRP3, IL‐18, and APACHE II score. (A) Correlation analysis between NLRP3 and APACHE II scores. (B) Correlation analysis between IL‐18 and APACHE II scores.

Pearson correlation analysis was conducted to estimate the relationship between the levels of NLRP3 and IL‐18 and the APACHE II score in patients with SA (Figure 5). Our data indicated that the expressions of NLRP3 and IL‐18 in PBMCs were positively correlated with the APACHE II score level, respectively (*r* = 0.5653, *p* < 0.001; *r* = 0.5385, *p* < 0.001).

### 3.5 NLRP3 and IL‐18 Could Serve As Sensitive Biomarkers for the Diagnosis of Sa‐Aki

**Figure 6:**
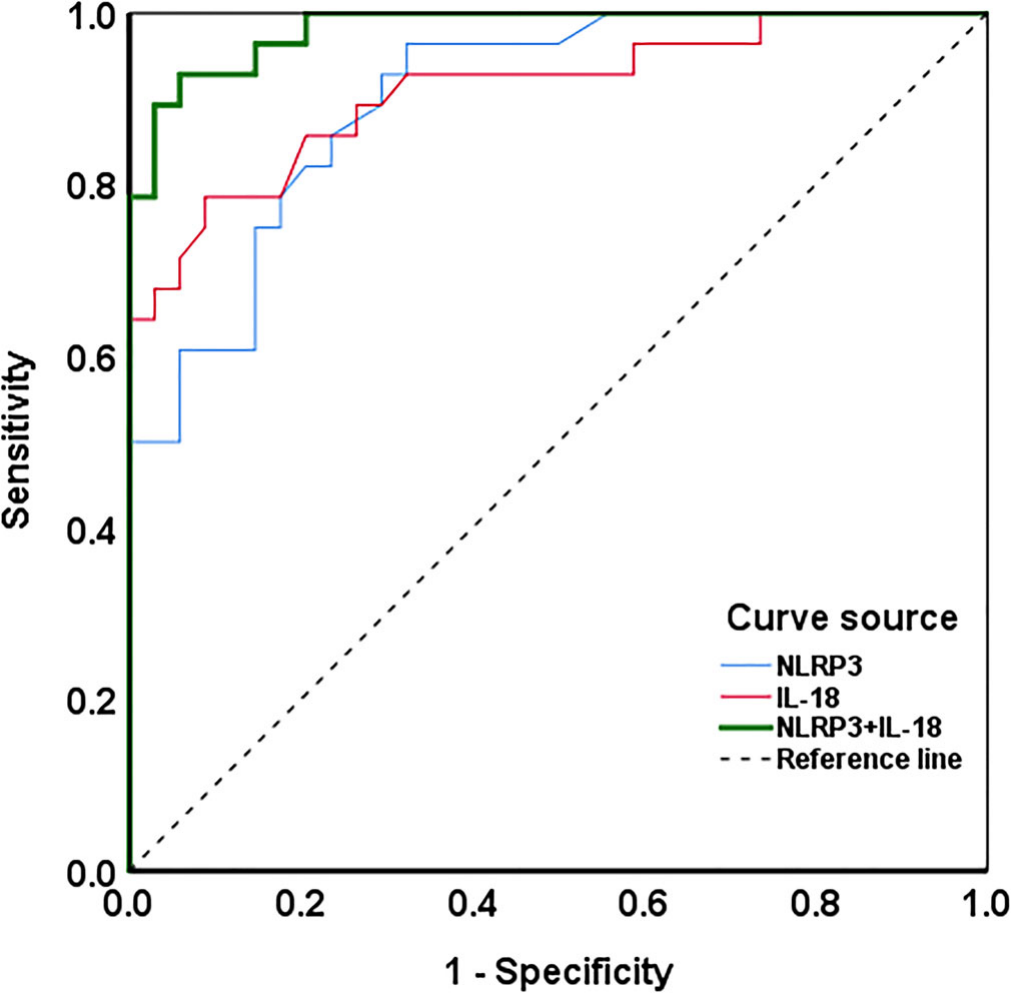
Receiver operating curves for NLRP3 and IL‐18 predict SA‐AKI risk.

To further investigate their utility in predicting the risk of AKI in SA patients, we conducted an ROC curve analysis. Figure 6 presents the ROC curve, while Table 3 details the diagnostic indicators for each biomarker. These include the area under the curve (AUC), sensitivity, specificity, positive predictive value (PPV), negative predictive value (NPV), and cutoff value. NLRP3 showed an AUC of 0.901 (95% confidence interval [CI]: 0.828–0.973), a sensitivity of 96.40%, and a specificity of 68.00%, yielding a PPV of 71.05% and an NPV of 95.83%. IL‐18 demonstrated an AUC of 0.910 (95% CI: 0.834–0.985), with a sensitivity of 78.60% and a specificity of 91.00%, translating to a PPV of 88.00% and an NPV of 83.78%. The combined AUC for NLRP3 and IL‐18 was 0.982 (95% CI: 0.958–1.000), with sensitivity and specificity improving to 85.70% and 94.00%, respectively, and predictive values reaching a PPV of 92.31% and an NPV of 88.89%. The determined cutoff values were 1.65 for NLRP3 and 2.21 for IL‐18. These results indicate the potential of NLRP3 and IL‐18 in PBMCs as diagnostic biomarkers for SA‐AKI.

**Table 3:** Diagnostic performance of NLRP3 and IL‐18 for SA‐AKI.

Biomarker	Sensitivity (%)	Specificity (%)	PPV (%)	NPV (%)	Cutoff value	AUC (95% CI)
NLRP3	96.40	68.00	71.05	95.83	1.65	0.901 (0.828–0.973)
IL‐18	78.60	91.00	88.00	83.78	2.21	0.910 (0.834–0.985)
NLRP3 + IL‐18	85.70	94.00	92.31	88.89	4.30	0.982 (0.958–1.000)

Abbreviations: 95% CI, 95% confidence interval; AUC, area under the curve; NPV, negative predictive value; PPV, positive predictive value.

## 4 Discussion

AKI is one of the most serious complications of sepsis. SA‐AKI has a high mortality rate despite the use of CRRT and other advanced life support techniques. Therefore, predicting the occurrence and prognosis of SA‐AKI has become an important clinical issue. Some researchers have studied the value of common indicators such as serum creatinine, BUN, and APACHE II in predicting the occurrence of SA‐AKI. However, the sensitivity and specificity of these indicators are low [10]. Therefore, biomarkers with high sensitivity and specificity are needed to identify the occurrence of SA‐AKI.

To find new biomarkers, we performed DEGs analysis, KEGG analysis, and GO analysis on the data set GSE61739. On the one hand, severe infection can lead to an unbalanced inflammatory response in SA‐AKI patients [6]. Furthermore, the results of a secondary analysis of the GSE61739 data set showed that NLRP3 and IL‐18 were upregulated in the kidney tissues of AKI patients compared with non‐AKI patients.

The NLRP3 inflammasome is a cytoplasmic multiprotein complex whose downstream products include IL‐18 and IL‐1β. When the body is infected or injured, the NLRP3 inflammasome can be activated, leading to the production of downstream inflammatory mediators. Studies have shown that the activation of NLRP3 inflammasome can promote SA‐AKI [11, 12]. Application of dexmedetomidine to AKI rats can reduce the activation of NLRP3 inflammasome and the expression level of IL‐18, enhance autophagy, and protect the kidney [13]. NLRP3 is expressed in monocytes and macrophages, and its activation is more pronounced in monocytes when stimulated [14]. The expression level of NLRP3 in PBMCs of patients with gouty nephropathy was significantly increased, and NLRP3 was correlated with the progression of gouty nephropathy [15]. The expression level of NLRP3 in PBMCs can reflect the intensity of the inflammatory response. However, the level of NLRP3 and its regulation of downstream signaling pathways in PBMCs of SA‐AKI patients are still unclear. Therefore, we chose NLRP3 and IL‐18 for clinical validation.

In this study, we measured the levels of NLRP3 and IL‐18 in PBMCs of different groups of patients. The results showed that the mRNA levels of NLRP3 and IL‐18 were significantly increased in sepsis patients compared with healthy controls. In addition, compared with the non‐AKI group, the levels of NLRP3 and IL‐18 were further increased in SA‐AKI patients. It is speculated that this may be related to the involvement of NLRP3 and IL‐18 in the process of sepsis. As sepsis progresses, the levels of NLRP3 and IL‐18 also increase, further aggravating renal tubular damage and leading to progressive decline in renal function. It was also found that the levels of NLRP3 and IL‐18 were positively correlated with the APACHE II score, further confirming the importance of NLRP3 and IL‐18 in the pathogenesis of SA‐AKI. ROC curve analysis showed that NLRP3 and IL‐18 had high evaluation value in distinguishing SA‐AKI, and the AUC was the highest when combined.

We measured the levels of NLRP3 and IL‐18 in PBMCs without correcting for altered cell composition. Therefore, our results should be interpreted with caution, and future studies should consider correcting for cell composition. Additionally, it is important to emphasize that our study assessed mRNA levels of NLRP3 and IL‐18, which do not necessarily correlate accurately with protein levels. Thus, our results do not directly indicate increased protein production of these biomarkers. Future studies are needed to investigate the protein levels of NLRP3 and IL‐18 in SA‐AKI patients.

Based on our findings, we propose that NLRP3 and IL‐18 could serve as significant early diagnostic biomarkers for SA‐AKI. However, our findings apply only to patients with SA‐AKI and may not be applicable to other inflammatory diseases. Therefore, the application of IL‐18 and NLRP3 as biomarkers in other conditions remains uncertain and requires further investigation. By assessing the expression levels of these two biomarkers, clinicians may detect the onset of SA‐AKI at earlier stages, enabling timely intervention for patients. Furthermore, these biomarkers have the potential to act as effective indicators for monitoring disease progression and treatment response in SA‐AKI. However, due to the small sample size of this study, the next step needs to further expand the sample size and verify it through multi‐center cooperation.

In conclusion, NLRP3 and IL‐18 in PBMCs can serve as potential diagnostic biomarkers for SA‐AKI, and their combination has higher sensitivity.

## Author Contributions

Jing Zhou and Yibin Ye participated in the design of this study and wrote the first draft of the manuscript. Zhipeng Chen, Yong Liu, and Baozheng Wu performed the experiment and analyzed the data set. Haiping Huang was in charge of reviewing and editing. All authors have read and approved the final manuscript.

## Ethics Statement

The research protocol was approved by the Ethics Committee ofZhangzhou Affiliated Hospital of Fujian Medical University (approval number: 2021KYB017).

## Conflicts of Interest

The authors declare no conflicts of interest.

## Data Availability

The authors have nothing to report.
